# Identification and Analysis of the Blood lncRNA Signature for Liver Cirrhosis and Hepatocellular Carcinoma

**DOI:** 10.3389/fgene.2020.595699

**Published:** 2020-12-07

**Authors:** Qi Xia, Zheyue Shu, Ting Ye, Min Zhang

**Affiliations:** ^1^State Key Laboratory for Diagnosis and Treatment of Infectious Diseases, National Clinical Research Center for Infectious Diseases, Collaborative Innovation Center for Diagnosis and Treatment of Infectious Diseases, The First Affiliated Hospital, College of Medicine, Zhejiang University, Hangzhou, China; ^2^Key Laboratory for Biomedical Engineering of Ministry of Education, Zhejiang University, Hangzhou, China; ^3^Zhejiang University, Hangzhou, China; ^4^Division of Hepatobiliary and Pancreatic Surgery, Department of Surgery, The First Affiliated Hospital, College of Medicine, Zhejiang University, Hangzhou, China; ^5^Key Laboratory of Combined Multi-Organ Transplantation, Ministry of Public Health, Hangzhou, China

**Keywords:** hepatocellular carcinoma, hepatitis B virus, lncRNA, liver cirrhosis, support vector machine

## Abstract

As one of the most common malignant tumors, hepatocellular carcinoma (HCC) is the fifth major cause of cancer-associated mortality worldwide. In 90% of cases, HCC develops in the context of liver cirrhosis and chronic hepatitis B virus (HBV) infection is an important etiology for cirrhosis and HCC, accounting for 53% of all HCC cases. To understand the underlying mechanisms of the dynamic chain reactions from normal to HBV infection, from HBV infection to liver cirrhosis, from liver cirrhosis to HCC, we analyzed the blood lncRNA expression profiles from 38 healthy control samples, 45 chronic hepatitis B patients, 46 liver cirrhosis patients, and 46 HCC patients. Advanced machine-learning methods including Monte Carlo feature selection, incremental feature selection (IFS), and support vector machine (SVM) were applied to discover the signature associated with HCC progression and construct the prediction model. One hundred seventy-one key HCC progression-associated lncRNAs were identified and their overall accuracy was 0.823 as evaluated with leave-one-out cross validation (LOOCV). The accuracies of the lncRNA signature for healthy control, chronic hepatitis B, liver cirrhosis, and HCC were 0.895, 0.711, 0.870, and 0.826, respectively. The 171-lncRNA signature is not only useful for early detection and intervention of HCC, but also helpful for understanding the multistage tumorigenic processes of HCC.

## Introduction

As one of the most common malignant tumors and the fifth major cause of cancer deaths worldwide (Jemal et al., 2011), hepatocellular carcinoma (HCC) is typical of highly invasive and metastatic potential. Although much progress has been made in clinical and experimental studies in HCC, the 5-year survival rate of HCC sufferers is still very low due to its poor prognosis, frequent clinical recurrence, and metastasis (Madkhali et al., 2015). The most important risk factors for liver cancer are hepatitis B virus (HBV), hepatitis C virus (HCV), excessive drinking, and exposure to aflatoxin B1. The geographical variability and heterogeneity of the incidence of HCC is different from the distribution of HBV and HCV infections on a global scale (Liu and Kao, 2007; Petruzziello et al., 2016). Globally, HBV accounts for about 80% of virus-related HCC cases, especially in Africa and East Asia, where the incidence of HCC is the highest. In low-incidence HCC areas such as Western Europe and North America, HCV infection accounts for about 20% of the total number of HCCs. HBV seems to be mainly related to the development of HCC (Blachier et al., 2013; Mittal and El-Serag, 2013; Kew, 2014; Ozakyol, 2017). About 15–40% of chronically infected people develop severe sequelae, such as cirrhosis, liver failure, and liver cancer, and nearly 1 million people die each year due to complications related to HBV^1^.

Hepatitis B virus infection facilitates virus-induced immune response through releasing cytokines and genotoxic reactive oxygen species, which triggers hepatocyte necrosis and may eventually contribute to the development of carcinogenesis with the speed-up of the hepatocyte cell cycles and raised risk of genetic variation (Budhu and Wang, 2006). Therefore, suppression of viral replication via antiviral therapy appears to decrease the risk of cirrhosis and HCC (Liaw et al., 2004; Hosaka et al., 2013). HCC in the early phase can be effectively treated through liver transplantation, resection, or ablation, whereas the treatment strategies are very limited for advanced patients (Llovet, 2014). Accordingly, comprehensive approaches to identify and validate novel markers are needed so as to provide a new idea for the early diagnosis and exploration of therapeutic targets of HCC.

Recently, loads of dysregulated long non-coding RNAs (lncRNAs) have been confirmed in HCC tumor tissue via high-throughput sequencing techniques, some of which may serve as early diagnostic biomarkers or therapeutic targets for HCC (Huo et al., 2017). LncRNAs are a subclass of non-coding RNAs that are able to modulate gene expression and cancer-related signaling pathways. Sufficient evidence suggested that lncRNAs are correlated with HCC cell biological functions, such as cell proliferation, cell apoptosis, the epithelial–mesenchymal transition (EMT) process, cell invasion, and tumor metastasis, and eventually result in the occurrence and progression of HCC (Qiu et al., 2017). For example, upregulation of several lncRNAs, including LncTCF-7, DANCR, ZEB1-AS1, and EGFR-AS1, have proven to play crucial roles in HCC progression via the activation of EMT and Wnt/β-catenin signaling (Yuen et al., 2009; Wang et al., 2015; Qi et al., 2016; Yuan et al., 2016). Downregulated lncRNA H19 has been shown to be associated with HCC metastasis (Zhang et al., 2013). Despite the fact that increasing studies have reported dysregulated lncRNAs in HCC, most of corresponding mechanisms and potential functions remain unclear. Further explorations on the regulation of these dysregulated lncRNAs, their mechanisms, and their association with the etiology of HCC may facilitate us to find more specific and sensitive markers to control HCC.

To identify the lncRNA signature associated with HCC progression, we analyzed the blood lncRNA expression profiles of 38 healthy control samples, 45 chronic hepatitis B patients, 46 liver cirrhosis patients, and 46 HCC patients. Advanced machine-learning methods like support vector machine (SVM), Monte Carlo feature selection, and incremental feature selection (IFS) were implemented for identification of the HCC progression-associated signature and construction of the prediction model.

## Materials and Methods

### The lncRNA Expression Profiles of Patients From Different Tumorigenesis Stages

We downloaded the blood lncRNA expression profiles of 38 healthy control samples, 45 chronic hepatitis B patients, 46 liver cirrhosis patients, and 46 HCC sufferers from GSE78160 included in the Gene Expression Omnibus (GEO). Expression levels of 2,520 lncRNA probes were assessed by State Key Laboratory Human lncRNA array 2412 (GPL21494^2^) developed by State Key Laboratory of Oncology in South China, Sun Yat-sen University. We would like to compare the differences among different tumorigenesis stages of HCC.

### The Importance of lncRNAs Is Calculated Using the Monte Carlo Feature Selection Method

The Monte-Carlo feature selection (Draminski et al., 2008) was employed to identify the key HCC lncRNAs. It is a widely used method with excellent performance in finding key features (Chen et al., 2018b,c; Pan et al., 2019). Monte Carlo feature selection can evaluate the importance of a feature by considering the contribution of the feature to accurate classification through a series of decision trees. Three steps are included: First, it will randomly choose many feature subsets; then, on each feature subset, a tree classifier will be built; and last, based on these trees, a compressive feature importance score will be calculated (Chen et al., 2018a; Pan et al., 2018; Wang et al., 2018). The final feature-importance score will consider both the frequency of this feature being selected by a tree and how well the node of this feature on the tree can classify the samples.

To introduce the details of this algorithm, the total number of lncRNAs was represented by *d*, which was 2,520 in this study. Each time, *m* lncRNAs (*m* ≪ *d*) are chosen at random and a tree classifier *t* is trained and tested on the basis of the randomly divided patients in the training and the test groups. This procedure will repeat *s* times. At last, there will be a series of trees. On the basis of the times a lncRNA *g* selected through these trees and the contribution of this lncRNA *g* to the tree classification, the relative importance (RI) of the lncRNA *g* can be calculated as follows:

(1) $${\text{RI}}_{\text{g}}=\sum_{\tau =1}^{s\,t}{\left(w\,A\,c\,c\right)}^{u}\,\sum_{n_{\text{g}}\,\left(\tau \right)}\text{IG}\,\left(n_{\text{g}}\,\left(\tau \right)\right)\,{\left(\frac{\text{no}.\text{in}\,n_{\text{g}}\,\left(\tau \right)}{\text{no}.\text{in}\,\tau }\right)}^{v}$$

where *wAcc* refers to the weighted classification accuracy of the decision tree τ; IG(*n*_g_(τ)) represents the information gained by node *n*_g_(τ), a decision rule based on lncRNA *g* expression; (no.in*n*_g_(τ)) stands for the total number of patients in node *n*_g_(τ); (no.inτ) refers to the total number of the patients under the decision tree τ; and *u* and *v* represent the adjusted parameters.

With Eq. (1), all lncRNAs will have a RI score and they will be ranked in line with their importance. The Monte Carlo feature selection method was implemented using the dmLab software (Draminski et al., 2008) accessed at https://home.ipipan.waw.pl/m.draminski/mcfs.html.

### Optimization of the lncRNA Signature With IFS Method

To optimize the number of selected lncRNAs, the IFS method (Jiang et al., 2013; Li et al., 2014; Shu et al., 2014; Zhang et al., 2014; Huang et al., 2015; Zhang P.W. et al., 2015; Chen L. et al., 2017) was employed. IFS can help determine how many features should be chosen. It assesses the performance of a series of SVM classifiers using various numbers of lncRNAs from one lncRNA, two lncRNAs, three lncRNAs to more lncRNAs. The SVM was a widely used classifier that was wrapped into IFS to evaluate the classification performance of different lncRNA sets. The lncRNA combination that had the best performance will be selected. It made the selection procedure objective and the chosen signatures had optimal performance.

In this study, the SVM classifier was established using the R function svm from package e1071^3^ with default parameters (SVM-Type: C-classification; SVM-Kernel: radial; cost: 1) and the classification accuracy was assessed with the aid of the leave-one-out cross-validation (LOOCV) method and then used to represent the prediction performance.

## Results and Discussion

### The HCC Progression lncRNAs Identified With Machine Learning Methods

The lncRNA importance was evaluated with the Monte Carlo feature selection method. It reflected how well the expression level of this lncRNA can correctly classify the healthy control samples, chronic hepatitis B patients, liver cirrhosis patients, and HCC patients. The rank of this importance provided basis for further optimization.

**FIGURE 1:**
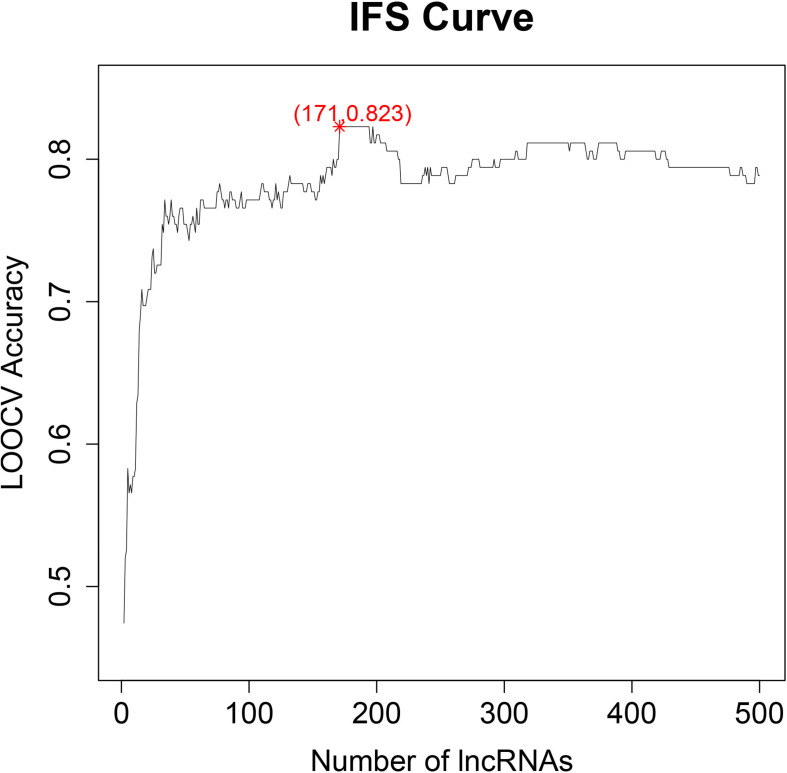
The IFS curve for key lncRNA selection. The *x*-axis is the number of lncRNAs used to build the SVM classifier. The *y*-axis is the prediction accuracy evaluated with LOOCV. When 171 lncRNAs were used, the accuracy was the highest as 0.823.

We optimized the top 500 ranked lncRNAs to 171 lncRNAs using the IFS method. Figure 1 shows the IFS curve in which the abscissa is the count of lncRNAs responsible for the establishment of the SVM classifier, and the vertical coordinate is the prediction accuracy assessed by LOOCV. The IFS curve peaked at (171, 0.823), which meant when 171 lncRNAs were used and the accuracy was the highest as 0.823. The accuracies of healthy control, chronic hepatitis B, liver cirrhosis, and HCC were 0.895, 0.711, 0.870, and 0.826, respectively. The 171 lncRNA probes are listed in Supplementary Table 1. The confusion matrix of the prediction performance using these 171 lncRNAs are listed in Table 1. It depicts that not only the overall accuracy, but also the accuracy of each progression stage.

**TABLE 1:** The confusion matrix of the prediction performance using 171 lncRNAs.

	Predicted stage 1	Predicted stage 2	Predicted stage 3	Predicted stage 4	Accuracy of each stage
Actual stage 1	34	3	0	1	34/(34 + 3 + 0 + 1) = 0.895
Actual stage 2	3	32	8	2	32/(3 + 32 + 8 + 2) = 0.711
Actual stage 3	0	5	40	1	40/(0 + 5 + 40 + 1) = 0.870
Actual stage 4	1	7	0	38	38/(1 + 7 + 0 + 38) = 0.826

*stage 1, healthy control; stage 2, chronic hepatitis B; stage 3, liver cirrhosis; stage 4, hepatocellular carcinoma.*

### The Biological Functions of the Identified lncRNAs

Since the lncRNA array was customized and did not have too much annotation, we blasted their sequences onto the lncRNA sequences in LNCipedia, version 5.0^4^ (Volders et al., 2018). Some of the identified lncRNAs were seen to be promising and may help understand the mechanisms underlying HCC tumorigenesis.

LUCAT1:20 ranked sixth in Supplementary Table 1. LUCAT1 (Lung Cancer Associated Transcript 1) was seen to be elevated in HCC tissue and cells relative to that in adjacent tissue, which was associated with pathological characteristics, such as tumor size, metastasis, and stage of HCC (Levine et al., 1988). Functional studies have unveiled the active role of LUCAT1 both in vitro and in vivo in potentiating the HCC tumor progression and metastasis (Levine et al., 1988; Gramantieri et al., 2018). LUCAT1 was also reported to bind to Annexin A2 (ANXA2) specifically, which is a phospholipids binding protein dependent on calcium and plays a vital role in the malignant behaviors of HCC cells with its expression elevated (Shi et al., 1993; Kohli et al., 2018). Zhang F. et al. (2015) indicated that the knock down of ANXA2 induced by shRNA inhibits hepatoma cell invasive and migratory capabilities and may hence become a therapeutic target for the molecular treatment of HCC in the future.

Lnc-RAP2B-5:1 ranked ninth in Supplementary Table 1. RAP2B, an Ras oncogene family (small GTP-binding proteins) member (Ohmstede et al., 1990), is a novel target of p53 regulating the cell pro-survival function (Qu et al., 2016). Increasing evidence suggests a critical role of RAP2B in the regulation of cytoskeletal organization, cell growth, cell proliferation, and other cellular processes (Uechi et al., 2009; Qu et al., 2016). Zhang et al. (2017) discovered the elevated expression of RAP2B in HCC tissue and cell lines, and revealed that the decreased RAP2B significantly downregulates the levels of p-FAK and MMP-2, and then inhibits HCC cell proliferation, invasion, and migration. Thus, Rap2B-targeted anticancer drugs are expected to become a novel therapy against cancer.

Lnc-FOXO1-2:3 ranked 15th in Supplementary Table 1. Forkhead Box Protein O1 (FOXO1), a member of the forkhead family, has been discovered to be dysregulated in multiple cancers including HCC, and it affects many cellular processes, like carcinogenesis, DNA damage repair, cell apoptosis, and tumor immunity (Huang and Tindall, 2007; Luo et al., 2016). It’s reported that higher FOXO1 significantly promotes replication and expression of HBV (Wang and Tian, 2017) and is related to a more favorable prognosis of HCC (Calvisi et al., 2009; Leung et al., 2015). EMT, a crucial process amid the occurrence of metastasis, is the principal reason for mortality in HCC (Papageorgis, 2015; Ye and Weinberg, 2015). Dong et al. (2017) found that FOXO1 is capable of reversing the EMT process through directly inhibiting transcription inducers like ZEB2, indicating the negative effect of FOXO1 on HCC cell proliferation and invasion. ZEB2 is reported to be upregulated in HCC cell-derived lung metastatic nodules, and its overexpression is responsible for HCC recurrence (Xia et al., 2014; Yang et al., 2015). Therefore, the enhancement of FOXO1 and the inhibition of EMT-related inducers like ZEB2 may have the potential to be applied in the clinical treatment of HCC with great value.

MALAT1:17 ranked 104th in Supplementary Table 1. As a long and highly conserved lncRNA widely expressed in different tissues (Zebisch et al., 2016), metastasis-associated lung adenocarcinoma transcription 1 (MALAT1) is regarded to be closely related to diverse cancer types, especially in the progression of HCC related to HBx (Jiang et al., 2014; Hou et al., 2017; He et al., 2019). The lncRNA−MALAT1 has been reported to be increased in HCC cell lines and it serves as a proto−oncogene amid the progression of HCC by means of activating the Wnt pathway and inducing the oncogenic splicing factor SRSF1 (Malakar et al., 2017). Furthermore, Liu et al. (2018) found that knockdown of MALAT1 suppresses the growth, migration, and motility of HCC cells by elevating miR-195, indicating that MALAT1 is an important player in the progression of HCC.

In addition to the aforementioned genes, lncRNAs including EPCAM (Yamashita et al., 2008), WDR5 (Cui et al., 2018), S1PR1 (Zhou et al., 2014), HMGA1 (Andreozzi et al., 2016), TGFBR2 (Chen Y.L. et al., 2017), CXCL12 (Semaan et al., 2017), and SENP2 (Shen et al., 2012) were also reported to participate in the pathogenesis of liver cirrhosis and HCC. Zhang et al. (2019) confirmed that silencing EPCAM can inhibit hepatic fibrosis and hepatic stellate cell proliferation in mice with alcoholic hepatitis through the PI3K/Akt/mTOR signaling pathway. Wenfang Tian et al. (2016) also found that WDR5 is an important epigenetic factor in the process of liver fibrosis. S1PR1 has been reported to be associated with cholestatic liver injury in early stage liver cancer and may be a potential target for the prevention of drug-induced cholestatic liver injury (Yang et al., 2017). HMGA1 was confirmed to be involved in the proliferation and invasion of HCC cells through the ilk/Akt/GSK3 signaling pathway (Liu et al., 2017). TGFBR2 is involved in regulating the regulation axis and aggravates liver fibrosis (Fu et al., 2020). The CXCL12/CXCR4 biological axis can inhibit the activation and migration of hepatic stellate cells in vitro and in vivo (Qin et al., 2018). SENP2 can reduce CCl_4_-induced liver fibrosis by promoting apoptosis and reversion of activated hepatic stellate cells (Bu et al., 2018).

Three lncRNAs mentioned earlier have been found to be associated with HBV. Studies have shown that MALAT1, WDR5, and CXCL12 are involved in the regulation of HCC induced by HBV through epigenetic mechanism. Bo He et al. (2019) found that interaction of lncRNA-MALAT1 and miR-124 regulates HBx-induced cancer stem cell properties through PI3K/Akt signaling. Weiwu Gao et al. found that WDR5 plays an important role in HBV-driven mouse hepatocyte proliferation and tumor growth (Gao et al., 2020). Chao Wang et al. (2017) found that HBx also upregulated the translocation of MDM2 into the nucleus and enhanced the transcriptional activity of CXCL12 and CXCR4.

Though the role of lncRNAs in HCC has been partially revealed, more large cohort studies and in-depth functional studies are still needed to validate the HCC lncRNA signature and to investigate the underlying mechanisms. In future research, we will focus on the biological functions of these lncRNAs in HBV infection, liver cirrhosis, and liver cancer, and further explore the molecular regulatory mechanism of these lncRNAs in cells to clarify the mechanism of lncRNAs and their important position in cells.

## Conclusion

Tumorigenesis is a multistage process. HBV infection is a trigger factor for liver cirrhosis and liver cirrhosis is a transition stage to HCC. The dynamic changes from normal to HBV infection, from HBV infection to liver cirrhosis, from liver cirrhosis to HCC, formed the chain reaction of tumorigenesis. We analyzed the blood lncRNA expression profiles of different HCC progression stages: healthy, chronic hepatitis B, liver cirrhosis patients, and HCC. A 171-lncRNA signature was identified with advanced machine-learning methods. These lncRNAs can help explain the mechanisms of HCC tumorigenesis. They can be used as biomarkers of HCC progression to monitor how bad the situation is and provide early detection and intervention of HCC.

## Data Availability Statement

The original contributions presented in the study are included in the article/Supplementary Material, further inquiries can be directed to the corresponding author/s.

## Author Contributions

QX, MZ, ZS, and TY all contributed to the study design and the final manuscript. They all gave the final approval of the version to be submitted. All authors read and approved the final manuscript.

## Conflict of Interest

The authors declare that the research was conducted in the absence of any commercial or financial relationships that could be construed as a potential conflict of interest.

## Footnotes

1 https://www.who.int/hepatitis/publications/global-hepatitis-report2017/en/

2 https://www.ncbi.nlm.nih.gov/geo/query/acc.cgi?acc=GPL21494

3 https://CRAN.R-project.org/package=e1071

4 https://lncipedia.org/

## Supplementary Material

Supplementary Table 1 The 171 lncRNA probes associated with HCC progression.
