# Corrigendum: Identification and Analysis of the Blood lncRNA Signature for Liver Cirrhosis and Hepatocellular Carcinoma

**DOI:** 10.3389/fgene.2021.692400

**Published:** 2021-07-01

**Authors:** Qi Xia, Zheyue Shu, Ting Ye, Min Zhang

**Affiliations:** ^1^State Key Laboratory for Diagnosis and Treatment of Infectious Diseases, National Clinical Research Center for Infectious Diseases, Collaborative Innovation Center for Diagnosis and Treatment of Infectious Diseases, The First Affiliated Hospital, College of Medicine, Zhejiang University, Hangzhou, China; ^2^Key Laboratory for Biomedical Engineering of Ministry of Education, Zhejiang University, Hangzhou, China; ^3^Zhejiang University, Hangzhou, China; ^4^Division of Hepatobiliary and Pancreatic Surgery, Department of Surgery, The First Affiliated Hospital, College of Medicine, Zhejiang University, Hangzhou, China; ^5^Key Laboratory of Combined Multi-Organ Transplantation, Ministry of Public Health, Hangzhou, China

**Keywords:** hepatocellular carcinoma, hepatitis B virus, lncRNA, liver cirrhosis, support vector machine

In the published article, there was an error in affiliation ^**^1^**^. Instead of “^**^Department of Infectious Disease, State Key Laboratory for Diagnosis and Treatment of Infectious Diseases, National Clinical Research Center for Infectious Diseases, Collaborative Innovation Center for Diagnosis and Treatment of Infectious Diseases, The First Affiliated Hospital, Zhejiang University School of Medicine, Hangzhou, China,^**^” it should be “^**^State Key Laboratory for Diagnosis and Treatment of Infectious Diseases, National Clinical Research Center for Infectious Diseases, Collaborative Innovation Center for Diagnosis and Treatment of Infectious Diseases, The First Affiliated Hospital, College of Medicine, Zhejiang University, Hangzhou, China.^**^”

Also, authors “Zheyue Shu” and “Min Zhang” were missing an affiliation: “^**^Division of Hepatobiliary and Pancreatic Surgery, Department of Surgery, The First Affiliated Hospital, College of Medicine, Zhejiang University, Hangzhou, China.^**^” Aforementioned corrections have been made and consequently, all affiliation numbers and order have been revised.

The authors apologize for this error and state that this does not change the scientific conclusions of the article in any way. The original article has been updated.

